# DNA mapping and kinetic modeling of the HrdB regulon in *Streptomyces coelicolor*

**DOI:** 10.1093/nar/gky1018

**Published:** 2018-10-29

**Authors:** Klára Šmídová, Alice Ziková, Jiří Pospíšil, Marek Schwarz, Jan Bobek, Jiri Vohradsky

**Affiliations:** 1 Institute of Microbiology, Academy of Sciences of the Czech Republic, 14220 Prague, Czechia; 2 First Faculty of Medicine, Institute of Immunology and Microbiology, Charles University, 12800 Prague, Czechia; 3 Chemistry Department, Faculty of Science, J. E. Purkinje University, 40096 Ústí nad Labem, Czechia

## Abstract

HrdB in streptomycetes is a principal sigma factor whose deletion is lethal. This is also the reason why its regulon has not been investigated so far. To overcome experimental obstacles, for investigating the HrdB regulon, we constructed a strain whose HrdB protein was tagged by an HA epitope. ChIP-seq experiment, done in 3 repeats, identified 2137 protein-coding genes organized in 337 operons, 75 small RNAs, 62 tRNAs, 6 rRNAs and 3 miscellaneous RNAs. Subsequent kinetic modeling of regulation of protein-coding genes with HrdB alone and with a complex of HrdB and a transcriptional cofactor RbpA, using gene expression time series, identified 1694 genes that were under their direct control. When using the HrdB–RbpA complex in the model, an increase of the model fidelity was found for 322 genes. Functional analysis revealed that HrdB controls the majority of gene groups essential for the primary metabolism and the vegetative growth. Particularly, almost all ribosomal protein-coding genes were found in the HrdB regulon. Analysis of promoter binding sites revealed binding motif at the −10 region and suggested the possible role of mono- or di-nucleotides upstream of the −10 element.

## INTRODUCTION

The gene expression in bacteria can be regulated at several levels; a key level being transcription by RNA polymerase holoenzyme (RNAP) and a specific sigma (σ) factor that recognizes the promoter sequence and allows transcription initiation (1,2). Different developmental complexities are controlled by a proportional number of sigma factors. Thus, the number of sigma 70 (σ^70^) family members varies from 1 in *Mycoplasma genitalium* (3) to about 66 in *Streptomyces coelicolor* (4). *Streptomyces* are gram-positive soil bacteria that undergo a complex multicellular development. Their growth starts with the germination of spores that develop into a vegetative mycelium of branching hyphae. The aerial hyphae are further dissected by sporulation septa into chains of uninucleoid spores. This is reflected by the fact that their genome was shown to encode more than 900 transcriptional regulators, among them the astonishing 66 sigma factors (5), the largest number found in a bacterium to date.

Promoter-recognition properties differ between the housekeeping sigma factor and a variable number of alternative sigma factors, which coordinate gene expression in response to various environmental signals. The housekeeping sigma factor in *Streptomyces* is HrdB (encoded by SCO5820 gene in *S. coelicolor*), whose presence is essential across the *Streptomyces* genus. HrdB, as well as HrdA, HrdC and HrdD sigma factors, is orthologous to RpoD (σ^70^) of *Escherichia coli* and σ^A^ of *Bacillus subtilis* and *Mycobacterium tuberculosis*, the principal sigma factors of these bacteria (6–10). However, neither HrdA-D regulons nor their role in developmental control have been studied so far in *Streptomyces*.

The RbpA protein (RNA polymerase binding protein A) is one of the transcriptional factors that are directly bound to sigma factor–RNAP (RNA polymerase) complex. RbpA binds to the β subunit of RNAP and modifies the enzyme core structure, which improves the affinity of RNAP to principal sigma factors HrdB and HrdA (11). These results are consistent with *Mycobacteria*, where RbpA is associated with sigma factor A or B (12,13). Deleting the *rbpA* gene is lethal for *Mycobacteria*, but not for *Streptomyces* (14). However, the absence of RbpA in *Streptomyces* leads to significantly impaired growth and increased sensitivity to rifampicin, an antibiotic which impedes transcription initiation (15–17). Interestingly, RbpA helps housekeeping sigma factors bind to RNAP but the *rbpA* gene itself is regulated by RNAP holoenzyme with alternative or stress response sigma factors (σ^R^ in *Streptomyces* and σ^E^ in *Mycobacteria*) and its expression is increased during oxidative stress (18,19). In summary, published results suggest that the complex of HrdB/RbpA with RNAP stimulates the transcription of genes controlled by HrdB, ensuring important cell functions.

In this paper, we focused on a systems level analysis of the HrdB regulon during the vegetative growth phase of *S. coelicolor*. Using insertional mutagenesis, the HrdB was adapted to carry an epitope tag which allowed the promoter binding to be determined by a ChIP-seq experiment. The resulting data were complemented with the gene expression kinetics analysis using data obtained from Gene Expression Omnibus (GEO). The data were used to evaluate the expression kinetic of the genes discovered by ChIP-seq. Computational modeling of gene expression kinetics were used, with a HrdB or HrdB–RbpA complex as regulators. The analysis helped us assess for which genes does binding of HrdB to the gene promoter actually results in transcriptional control.

## MATERIALS AND METHODS

### Strains and growth conditions

The strains used in our work are *S. coelicolor* A3(2), *E. coli* K-12 MG1655 (20) and derivatives from GM2929 (21). *Escherichia coli* BW25113/pIJ790 has a λ Red recombination system under the control of an arabinose inducible promoter and this strain was used to propagate the *S. coelicolor* cosmid. *Escherichia coli* ET12567/pUZ8002, which is a methylation-deficient strain for intergeneric conjugation with *S. coelicolor*. For the preparation of the epitope tagged mutant strain, *S. coelicolor* was cultivated on solid agar plates with MS medium (2% (w/v) mannitol, 2% (w/v) soya flour, 2% (w/v) bacterial agar in tap water) or DNA medium (2,3% (w/v) Difco nutrient agar) (22). Apramycin (50 µg/ml), chloramphenicol (25 µg/ml), kanamycin (50 µg/ml) or nalidixic acid (25 µg/ml) was added to the media when needed. The list of genetic material used is shown in Table 1. For ChIP-seq analysis, spores stocks were prepared by harvesting them from agar plates grown for 10 days. Following the procedure described in Nieselt *et al*. (23), we inoculated thawed spore stock (2,5*10∧9/ml CFU—typically 3 ml) to 100 ml 2YT medium (bacto tryptone, 16 g/l; bacto-yeast extract, 10 g/l; NaCl, 5 g/l) to 500 ml Erlenmeyer’s flask (customised to enable aeration) and germinated at 30°C for 5 h. The germinated spores were harvested by centrifugation (3200 × *g*, 15°C, 5 min) and resuspended in 5 ml ion-free water. Then they were inoculated into an 1.8l Na-glutamate medium (Na-glutamate, 61 g/l; glucose monohydrate, 44 g/l; MgSO_4_, 2.0 mM; Na_2_HPO_4_, 2.3 mM; KH_2_PO_4_, 2.3 mM) supplemented with 8 ml/l of trace element solution (ZnSO_4_ x 7 H_2_O, 0.1 g/l; FeSO_4_ × 7 H2O, 0.1 g/l; MnCl_2_ × 4 H2O, 0.1 g/l, CaCl_2_ × 6H_2_O,0,1 g/l, NaCl 0,1 g/l) (22) and 5.6 ml/l of TMS1 (FeSO_4_ × 7 H_2_O, 5 g/l; CuSO_4_ × 5 H_2_O, 390 mg/l; ZnSO_4_ × 7H_2_O, 440 mg/l; MnSO_4_ × H_2_O, 150 mg/l; Na2MoO4 × 2H_2_O, 10 mg/l; CoCl2 × 6 H_2_O, 20 mg/l; HCl, 50ml/l). Cultivation was conducted at 30°C at 250 RPM and pH of 7 was maintained during growth. Samples for the ChIP-seq analysis were collected after 22 h (equivalent to exponential phase) of growth (23).

**Table 1.** Strains, plasmids, cosmids and oligonucleotides used in this study

Strains	Genotype/comments	Reference/source
*S. coelicolor*		
A3(2) WT	SCP1- SCP2-	(4)
A3(2) *hrdB*-HA	HA tagged *hrdB* mutant strain:: *apr oriT* cassette	This study
*E. coli*		
ET12567	*dam, dcm, hsdM, hsdS, hsdR, cat, tet*	(21,25)
BW25113	K-12 derivative; Δ(*araD-araB*)*567* Δ *lacZ4787*(::*rrnB-4*) *lacIp-4000*(*lacI^Q^*), l*-rpoS369*(*Am*) *rph-1* K-12 derivative; Δ(*araD-araB*)*567* Δ *lacZ4787*(::*rrnB-4*) *lacIp-4000*(*lacI^Q^*), l*-rpoS369*(*Am*) *rph-1*	(20)
Plasmids/cosmids		
pIJ773	P1-FRT-*oriT-aac(3)IV*-FRT-P2 (plasmid template for amplification of the *apr oriT* cassette for ‘Redirect’ PCR-targeting)	(40)
pIJ790	λ-RED (*gam, bet, exo*), *cat, araC, rep101^ts^*	(40)
pUZ8002	*tra, neo*, RP4	(63)
St5B8	*carb, kan*	cosmid library University of Wales Swansea
St5B8 hrdB-HA	*carb, kan*, HA tagged *hrdB* mutant strain:: *apr oriT* cassette	This study
Oligonucleotides		
HrdB_HAtag_up	CACCCCTCGCGCTCGCAGGTGCTGCGCGACTACCTCGACTACCCATACGACGTCCCAGACTACGCTTAGATTCCGGGGATCCGTCGACC	
HrdB_HAtag_down	CGTCTGGTCGTACCGCCGGTCCGTACGGTCGGCTACGACTGTAGGCTGGAGCTGCTTC	

### Construction of epitope-tagged mutant strains

In order to insert the HA-tag to the *hrdB* gene in its native chromosomal position, we modified the mutagenesis procedure (24) as follows: the nucleic acid sequence of the HA tag (YPYDVPDYA) was optimized for the codon usage in *S. coelicolor* (TAC CCG TAC GAT GTG CCG GAT TAC GCG). A gene cassette containing FRT flanking regions, apramycin resistance marker and oriT was amplified from plasmid pIJ773 as described (24), cut by EcoRI and HindIII restriction enzymes and used as a polymerase chain raection (PCR) template. The purified PCR product was then electroporated into *E. coli* BW25113/pIJ790 containing the *S. coelicolor* cosmid 2StK8. The cells were then cultivated at 37°C for 1 h in 1 ml LB. The culture was centrifuged for 15 s, at 10 000 *g* and spread onto LB agar with apramycin (50 μg/ml). The cosmid with the inserted cassette was then transformed into the methylation-deficient *E. coli* ET12567/pUZ8002 and the resulting strain was conjugated with *S. coelicolor* A3(2) (25). Final mutants were selected on MS medium containing apramycin, kanamycin and nalidixic acid (22). Double cross-over exconjugants (kanamycin sensitive, apramycin resistant) were selected. Chromosomal DNA was then isolated and the cassette's integration into the chromosome was confirmed by PCR and sequencing.

### Chip-seq

#### Sample preparation

ChIP-seq analysis was done as was described previously with minor modifications (26). Briefly, after 22 h of growth, cells were incubated for 30 min with 1% formaldehyde (CH_2_O 36,5%–38% Sigma Aldrich). Crosslink reactions were stopped with prechilled 2 M glycine for 5 min at room temperature. Cells were harvested by centrifugation at 4°C and washed five times with 1 × phosphate-buffered saline. The pellet was stored at −80°C prior further processing. Subsequently, the pellet was resuspended in RIPA buffer (SDS, 0.1%; sodium deoxycholate, 0.5%; Triton X-100, 0.5%; 1 mM; NaCl, 150 mM; Tris, pH 8, 50 mM; Proteases inhibitor-Complete, Mini, ethylenediaminetetraacetic acid (EDTA)-free, Roche, 10 nM) and sonicated 6 × 15-s, amplitude 0.5, on ice (Hielscher sonicator, UP 200S). The DNA fragments lengths were determined by agarose gel electrophoresis and were shown to be in the desired range of 200–500 bp.

The cell lysate was centrifuged at 4°C, 20 000 × *g*, 20 min. Supernatant was collected and precleared for 2 h at 4°C by pre-equilibrated Protein-G Plus Agarose (Santa Cruz sc2002) in RIPA buffer. The concentration of proteins was measured and 2 mg of total proteins in lysate were added to 2 μg of anti-HA high affinity antibody (Roche, 11867423001) and incubated for 16 h at 4°C. Protein-G Plus Agarose was added and incubated for 6 h at 4°C.

Samples were washed three times with RIPA buffer, four times with WASH buffer (Triton X-100, 0.5%; Sodium deoxycholate, 0.4%; LiCl, 0.5 M; Tris, pH 8.5, 100 mM), twice with RIPA buffer a second time, twice with TE buffer (EDTA, 10 mM; Tris, pH 8, 10 mM). Subsequently, elution was achieved using Elution buffer (SDS, 1%; EDTA, 10 mM; Tris, pH 8, 50 mM).

Decrosslink reactions were done by addition of 200 mM NaCl and Proteinase K at 60°C overnight. Finally, DNA was purified by NucleoSpin gDNA Clean-up (Macherey-Nagel). The negative control was the wild-type strain of *S. coelicolor* that was processed in the same way.

#### Library preparation and sequencing

Libraries were prepared with NEBNext^®^ Ultra™ II DNA Library Prep Kit for Illumina^®^ (NEB #E7645), and all material was used as input. The adaptors were diluted 1:30 times, and 17 PCR cycles were performed. Libraries were measured with Qubit DNA High sensitivity assay, and afterward loaded on Agilent Bioanalyzer 2100 with DNA 1000 kit. Samples were pooled equimolar before sequencing, and the pool was measured with Qubit DNA high sensitivity kit. The pool was clustered on cBot. Before clustering samples were diluted and denatured following Illumina recommendations (cBot system guide). Samples were diluted to 3.5 nM as final concentration for clustering. Libraries were sequenced on HiSeq 3000/4000 (HiSeq 4000 system guide). Run was performed with a 50 cycles kit, and the mode was 50 bp single read (51 bp read 1+ 7 bp index read). The samples were sequenced at EMBL-Gene Core, Germany.

#### Data analysis

Raw ChIP-seq data were processed in Chipster (https://chipster.csc.fi/) ran on the server of the Metacentrum (https://www.metacentrum.cz/).

Three samples and two controls fastq data files were analyzed (ChIP-seq data have been deposited in the ArrayExpress database at EMBL-EBI (www.ebi.ac.uk/arrayexpress) under accession number E-MTAB-6926). Quality control of the sequences was done individually for each file using FastQC. According to the report, data were trimmed left by 5 nt. Sample files and control files were concatenated for further processing. Genome sequence annotation file (.gtf) and the sequence file (fasta) were downloaded from Ensembl database (ftp://ftp.ensemblgenomes.org/pub/bacteria/release-37/fasta/bacteria_0_collection/streptomyces_coelicolor_a3_2_/dna/). The data were aligned using Bowtie2 algorithm. Peaks in the aligned sequence were identified using MACS2. Peaks were mapped to the *S. coelicolor* reference genome (GenBank: NC_003888) and were inspected in Genome browser. Corresponding genes and their operons were identified by selecting statistically significant peaks (*P* < 0.05) with fold enrichment ≥ 2. Target genes were identified according to peak summits in ≤ 300 bp upstream of an annotated start codon of the respective gene. Operon categorization were done using operon prediction approach published in (27). Altogether 2147 genes were identified.

### Modeling gene expression profiles

To model the possible regulatory effects of HrdB we used the Genexpi tool and associated workflow (28) with minor additions. In particular, the expression of all genes was first smoothed with a B-spline and the smoothed expression of putative targets was modeled as an ordinary differential equation (ODE). A maximum a-posteriori estimate of the parameters of the ODE was determined with simulated annealing. We tried to fit three models of increasing complexity to the time series of the target gene expression. After each model was applied, the results were filtered by fit quality. Only genes that did not fit sufficiently well with a simpler model were tested with a more complex model. The three models were—the constant synthesis model, direct model and cooperative model. The constant synthesis model assumes that the synthesis rate of the target is constant over time:

(1) \begin{equation*}\frac{{dx}}{{dt}} = {k_1}\ - {k_2}x\end{equation*}

Here, *x* is the expression of the target gene as a function of time, *k_1_*and *k_2_* are mRNA synthesis and degradation rate constants. When a gene is fit by the constant synthesis model, it does not necessarily mean that it is not regulated by HrdB, only that its synthesis is not affected by concentrations changes of HrdB observed in the experiment.

The direct model assumes that the target gene is regulated solely by HrdB:

(2) \begin{equation*}\frac{{dx}}{{dt}} = {k_1}\ \frac{1}{{1 + {e^{ - \left( {wy\ + \ b} \right)}}}} - {k_2}x\end{equation*}

Here, *y* is the smoothed expression of the regulator (HrdB) as a function of time, *k_1_* is related to the maximum possible synthesis, *w* is the regulatory weight, *b* is bias (inversely related to the regulatory influence that saturates the synthesis of the mRNA) and *k_2_* is mRNA decay rate constant. For further details on those two models and the optimization procedure, see (28).

The cooperative model assumes that the target is regulated by a complex of HrdB and RbpA. This is a slight extension of the direct model and it is a novel contribution of this paper. The model follows Equation 2, but the HrdB + RbpA complex is considered as a regulator (*y*). The concentration of the complex is in turn determined by the concentrations of its constituents and an equilibrium constant *q*:

(3) \begin{equation*}q\ = \frac{{{y_A}{y_B}}}{{{y_{AB}}}}\ = \frac{{\left[ {HrdB} \right]\left[ {RbpA} \right]}}{{\left[ {HrdB-RbpA} \right]}}\ \end{equation*}

Solving for ${y_{AB}}$we get

(4) \begin{eqnarray*}{y_{AB}}= y = \frac{1}{2} \left( {{y_A} + {y_B} + q - \sqrt {{{\left( {{y_A} - {y_B}} \right)}^2} + 2q\left( {{y_A} + {y_B}} \right) + {q^2}} } \right)\end{eqnarray*}}

In practice, this means that ${y_{AB}}$is interpolated between 0 (for $q \to \infty$) and $min{\{ {{y_A},{y_B}} \}_{}}$ (for $q \to 0$) and it is used as a regulator (y) in Equation 2. The equilibrium constant *q* becomes another parameter of the ODE that is optimized jointly with the other parameters of Equation 2. Source code for fitting all of the models is available at https://github.com/cas-bioinf/genexpi.

### Gene class enrichment analysis

Gene functional categories were obtained from the Sanger database for *S. coelicolor* A3(2) (ftp://ftp.sanger.ac.uk/pub/project/pathogens/S_coelicolor/classwise.txt), where each gene identified by its SCO number is characterised hierarchically into four categories –protein, Sanger_database_x00, Sanger_database_xx0, Sanger_database_xxx. Genes in this work were characterized by the category Sanger_database_xx0, (this table is repeated in each sheet in supplementary file 1).

### Binding motif search

The binding motif search was based on known transcription start sites (TSS) for *S. coelicolor* published by Jeong *et al*. (29).

#### Promoter region location

For the genes that were assigned to individual ChIP-seq peaks we found those for which the TSS was known from the Jeong's paper (1723 sites). From this set, we selected only genes with TSS assigned to the category ‘primary’. Final set contained 1048 TSS, (filtered TSS).

#### Motif discovery

Based on known genome loci of the filtered TSS we extracted two locations, 20 bp upstream to 0 relative to each TSS for −10 region and −40 to −25 bp upstream relative to −35 region. The motif search was performed with MEME software (http://meme-suite.org/) using two approaches. Method 1 (same as Jeong’s *et al*. used) uses MEME with -dna -oops parameters, meaning that one motif site from each sequence (here promoter region) contribute to resulting motif. Final motif is then obtained by filtering for motif sites with *P*-value < 0.05. Method 2 uses MEME with -dna -zoops parameters, meaning that 0 or 1 motif sites are expected per sequence and only motif sites with *P*-value < 0.05 contribute to final motif. Discovered motifs were compared with the published ones found by Jeong *et al*. The motif positions numbering was relative to TSS described in the Jeong’s paper, and motifs shown are drawn from aligned sequences.

#### Presence of G or GG immediately prior to located motif

According to literature (30), the presence of G or GG motif prior to −10 region stabilizes the binding of the transcription factor holoenzyme. We therefore inspected the −14 and −13 regions for occurrence of GG or G. As a source data we took locations for motifs with *P* < 0.05 as found with MEME by the method 2 (see above) (954 sites). Then we considered our motif to be TANNNT and we extracted exactly 2 nucleotides ahead of that motif (referenced as −14 and −13). Then we grouped the motif sites by presence of G nucleotide into three groups (GG—both positions contain G, G—one of positions contains G (but not both), noG—no position contain G). The expected frequency of dinucleotides GG was calculated as *f_GG_ = count(GG)/(length(genome)-1)*, where count(GG) means number of all occurrences of GG dinucleotide in genome. The expected number of sites having GG randomly was N = f_GG_*number of sites, where *f_GG_* is frequency of dinucleotide GG in genome, and *number of sites* represent number of sites analyzed.

## RESULTS

### Identification of *in vivo* HrdB-binding DNA regions by ChIP-seq

To study the binding of HrdB to the *S. coelicol*or genome, we tagged HrdB with an HA tag and performed a ChIP-seq analysis to reveal the binding regions of HrdB *in vivo*. For this, we constructed an HA-tagged HrdB strain using insertional mutagenesis. Then, the HA-epitope tag helped us to isolate the HrdB-binding sites by chromatin immunoprecipitation with a specific anti-HA antibody. Cells were cultivated in the medium identical to the one defined by Nieselt *et al*. (23), whose gene expression database we exploited for the gene expression kinetics analysis. The ChIP sequencing data were analyzed using Chipster bioinformatics pipeline (see Materials and Methods). Altogether we detected 1245 HrdB-binding regions (0.05 significant and fold enrichment ≥ 2).

We annotated the target genes according to the location of the peak summits. For peak summits located in ≤300 bp upstream of annotated start codon, HrdB was considered to regulate the respective gene. Thus, 1599 genes were identified using this criterion. We also incorporated genes of putative operons predicted according to the operon prediction approach as published in (27) and we identified 337 operons consisting of 2137 protein-coding genes, 75 small RNAs, 62 tRNAs, 6 rRNAs and 3 miscellaneous RNAs (list of all genes is given in Supplementary File 2).

### Protein coding genes data analysis

#### Gene expression time series

**Figure 1.**
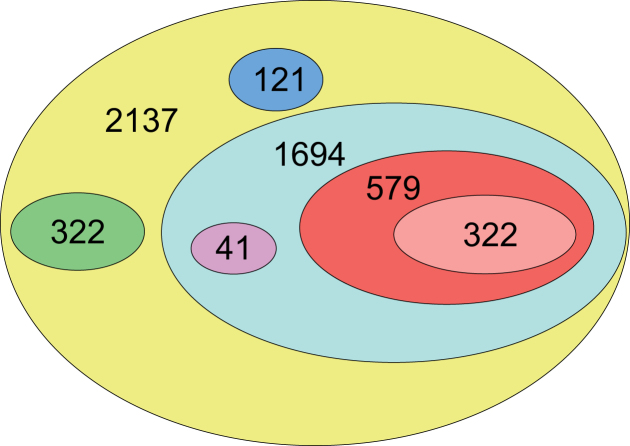
Schematic representation of number of genes in different groups distributed according to the modeling results. A total of 2137 genes were found by ChIP-seq and were present in the time series database. A total of 322 genes exhibited a constitutive rate of expression. A total of 121 genes could not be modeled at all. The kinetics of 1694 genes was modeled with HrdB or a HrdB–RbpA complex. Of them, 41 genes could be modeled by the complex only. A total of 579 genes gave 10% better fit with the complex as regulator and 322 genes gave a 20% better fit with the complex as regulator.

The 2137 found genes were searched in the gene expression database published by Nieselt *et al*. (23) downloaded from GEO with accession number GSE18489. The time series contain gene expression measurements in 32 time points without replicates (20, 21, 22, 23, 24, 26, 27, 28, 29, 30, 31, 32, 33, 34, 35, 36, 37, 38, 39, 40, 41, 42, 43, 44, 46, 48, 50, 52, 54, 56, 58, 60 h). The data in individual time points were normalized to have the same mean of the distribution of expression values. The data that had been published in log2 scale were, for the following analysis, exponentiated to give original microarrays reads. As the Nieselts experiments were run without replicates, the variance of the expression profiles is very high. In order to find the trend in expression, the time series were splined using piecewise cubic spline with four anchor points as defined in Lundgren’s procedure (31). Of the 2147 genes found by the ChIP-seq analysis results, gene expression time series were found in the Nieselts database for 2137 genes (Figure 1).

**Figure 2.**
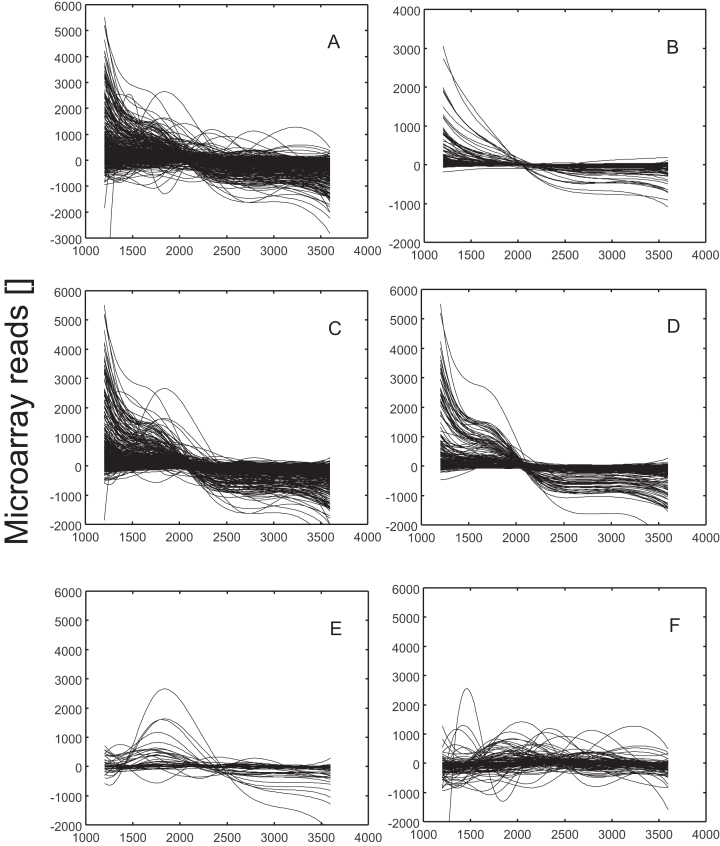
Splined expression profiles of genes selected by various models. (**A**) All genes of the HrdB regulon as found by ChIP-seq, (**B**) genes with constant rate of transcription, (**C**) genes modeled with HrdB or HrdB–RbpA complex, (**D**) genes modeled 20% better with HrdB–RbpA complex than with HrdB alone, (**E**) genes modeled exclusively by the complex HrdB–RbpA, (**F**) genes not modeled by any of the models used. The profiles were splined and normalized to have zero mean. Expression profiles of HrdB and RbpA, used for the computational modeling, are shown in Figure 3. The data were obtained from GEO, accession number GSE18489

**Figure 3.**
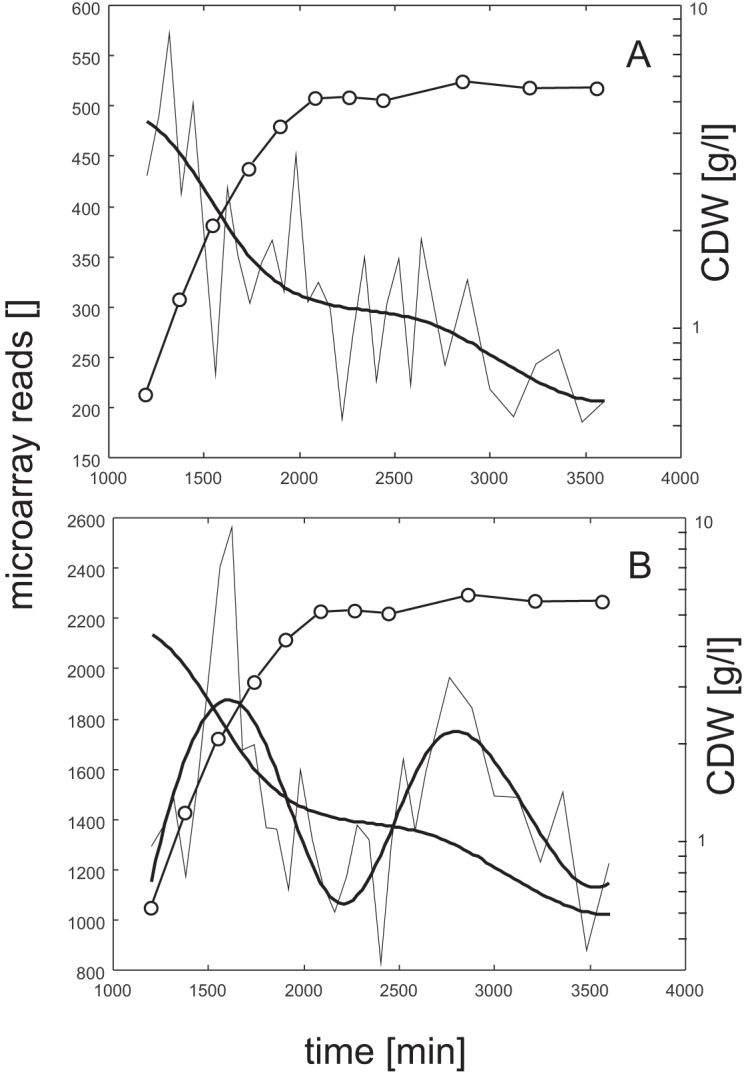
Expression profile of HrdB (**A**) and RbpA (**B**), splined and measured together with the growth curve. The data were obtained from GEO, accession number GSE18489 and (23).

Previously published data suggest the important function of RbpA in the initial step of transcription (see ‘Introduction’ section). For this reason, we included expression data of the *rbpA* gene into the model. The expression profiles of the 2137 genes were subjected to kinetic analysis using three models—constitutive rate of expression, expression controlled by HrdB, and expression controlled by the HrdB–RbpA complex as described in Methods (expression profiles of all regulated genes are shown in Figure 2A). Expression profiles of HrdB and RbpA mRNA, used for the computational modeling, are shown in Figure 3.

#### Genes with constitutive rate of expression

The meaning of constitutive rate of expression here is that the expression profile of the mRNA corresponding to the controlled gene is independent of the expression profile of the sigma factor or the complex. The rate of the mRNA accumulation of these genes was in this case the result of the difference between a constant and a degradation term. It is possible to model such profiles with Equation 1. Constraints for the goodness of fit were met for this model by 322 genes (Supplementary File 1, sheet ‘fit comparison’). Expression profiles of these genes, normalized to have a mean of zero, are shown in Figure 2B.

Gene class enrichment analysis (Supplementary File 1, sheet ‘func no control’) showed that, except for the genes coding for amino acid biosynthesis (10 of 123 total), no specific enrichment was found in the functional groups. Even the AA biosynthesis group did not contain specific gene families. It can be concluded that this group contained an unspecific mixture of genes.

#### Genes controlled by HrdB or the complex HrdB–RbpA

(Supplementary File 1 sheets ‘HrdB or complex controlled’ and ‘func hrdB or hrdBrbpA’).

The remaining 1815 genes were modeled with HrdB or the HrdB–RbpA complex as regulators. A total of 121 of them could not be fitted with HrdB, nor the HrdB–RbpA complex, resulting in 1694 genes that were controlled either by HrdB or the complex (Figure 2C). Of these 1694 genes, 41 could be fitted only with the HrdB–RbpA complex (Figure 2E). When comparing the fit between HrdB and HrdB–RbpA complex, 10% better fit using the complex was achieved for 579 (34%) genes, 20% better fit for 322 (19%) genes (Figure 2D).

In order to estimate the false positive rate of the model predictions, we randomized the HrdB expression profile 20 times. For each of the randomized profile we optimized the parameters for each gene of the HrdB regulon in the same way as for the original analysis (see ‘Materials and Methods’ section). Then for each individual optimization we evaluated the fit quality. About 56% of the regulon profiles modeled with the randomized time series satisfied the goodness of fit criteria. For about a half of them (22% of all), the parameter *w* was close to zero (<0.0001), i.e. indicating no control that have to be excluded. Altogether, 34% of the gene expression time series could be fitted by the randomized HrdB expression profile. The number represents the maximal estimate of the possible false positives. The degree of accuracy corresponds to the degree of accuracy of the measured time series and is comparable with the results of similar analyses (28).

Gene class enrichment analysis of the 1694 genes showed that the highest set abundance among all the genes was found in the categories Chromosome replication (2.9×, 5 of 8 total, mostly DNA polymerase subunits), 3.5× higher in Nucleotide biosynthesis group (23 of 30 total, purine/pyrimidine metabolism) and 3.2× higher for the category ribosome constituents (47 of 67 total) formed mostly by ribosomal proteins. Also sigma factors SCO0037, 0038, 0414, 1723 (sigK), 2639, 3202 (hrdD), 3709, 4336, 4409, 5621 (whiG), 6996, 7278 and anti-sigma factor SCO0599 (rsbA), anti-sigma factor antagonists SCO0869, 3549 (bldG), 3692, 4027 and anti-anti-sigma factor SCO3067 were found.

Detailed description of genes as members of selected functional groups essential for cell function are summarized in the following paragraphs. Genes mentioned below as members of the HrdB regulon represent genes found by the model to be controlled by HrdB and correspond to the cyan region in Figure 1. Division to functional groups and subgroups can be found for all genes in the Supplementary File 1.

##### Nucleotide biosynthesis

The nucleotide biosynthesis group consisted of the subgroups ‘purine biosynthesis’ (21 genes) and ‘pyrimidine biosynthesis’ (9 genes). We identified nearly 77% of all genes from this group within the HrdB modeled regulon and it was the most abundant group of all. In the ‘purine biosynthesis’ subgroup we found 17 genes (81%)—prsA (SCO0782), purB (SCO1254), guaB2 (SCO1461), purE (SCO3059), purK (SCO3060), prsA2 (SCO3123), purA (SCO3629), SCO3677, purQ (SCO4078), purl (SCO4079), purF (SCO4086), purM (SCO4087), adk (SCO723), guaB (SCO4770), SCO4771, purN (SCO4813), purH (SCO4814). In the ‘pyrimidine biosynthesis’ subgroup we found six genes (67%)—pyrB (SCO1487), carB (SCO1483), pyrAA (SCO1484), pyrC (SCO1486), pyrF (SCO1481), cmk (SCO1760).

##### Ribosome constituents and translation

The ‘ribosome constituents’ group comprised mostly genes coding for 30S and 50S ribosomal subunits and also genes responsible for ribosome maturation and modification such as 16S rRNA-processing protein RimM (SCO5593), tRNA pseudouridine synthase B (SCO5709) and pseudouridine synthase (SCO1768). HrdB regulated 70% of all ribosome constituents coded in the *Streptomyces* genome. The ‘Ribosome constituent’ group consists of two subgroups—‘ribosomal proteins’, containing 62 ribosomal proteins and a second ‘ribosomes—maturation and modification’, consisting of five genes (in the HrdB modeled regulon, we identified three genes, 60%).

Additional genes essential for translation were present in the group ‘Proteins—translation and modification’, where HrdB regulated 22 genes of total 36 (61%). From this group we identified in the HrdB modeled regulon elongation factors (Tu (SCO1321, SCO4662), P (SCO1491), Ts (SCO5625), G (SCO1528, SCO4661)), translation initiation factor (IF-1 (SCO4725), IF-3 (SCO1600), signal peptidases (SCO5596-SCO5599), ribosome-binding factor A (SCO5708), signal recognition particle protein (SCO5586) (in the ‘others’ group we also identified srp RNA consisting of 4,5S rRNA), peptidyl-prolyl cis-trans isomerase (SCO1510), peptidyl-prolyl isomerase (SCO5939)).

##### Chromosome replication

The functional group ‘chromosome replication’ consists of eight genes altogether, and we found five of them in the HrdB modeled regulon. These were: dnaE—DNA polymerase III subunit alpha (SCO2064), dnaN—DNA polymerase III subunit beta (SCO3878), dnaZ—DNA polymerase III subunit gamma and tau (SCO4067), dnaA—chromosomal replication initiation factor (SCO3879) and dnaB—replicative DNA helicase (SCO3911).

The remaining DNA replication proteins were present in the group ‘2.2.3 DNA replication, repair, restriction modification system’, where HrdB regulated 21 genes of total 85 (around 25%). The HrdB regulon contained genes such as DNA helicases (SCO1167, SCO3550, SCO4092, SCO5815), DNA topoisomerases (I-SCO3543, IV subunit beta—SCO5822), subunits of DNA gyrase (A—SCO3873, B—SCO3874), DNA ligase SCO6707).

##### Transcription

In the HrdB regulon we revealed almost 26 genes that participate in the transcription process. These were: RNA polymerases subunits, transcription termination factor Rho, transcription elongation factor NusA and GreA, transcription antitermination protein NusB and mainly sigma factors including WhiG, SigK, HrdD and anti-sigma factors and their antagonists.

##### Cell division

In the HrdB modeled regulon we also found genes coding for major components of the cell division machinery, including ftsZ, ftsI, ftsH-like protein, SCO2968 (similar to ftsX of *M. tuberculosis*), septum determining protein.

#### Genes under putative control of the HrdB–RbpA complex

(Supplementary File 1, sheets ‘hrdB or complex controlled’, ‘func 20% better fit hrdBrbpA’).

The published data suggest that RbpA facilitates the transcription of HrdB controlled genes (see ‘Introduction’ section) in *Streptomyces*. We therefore inspected the genes that were successfully modeled with HrdB in order to discover if there is any improvement of fit when using the HrdB–RbpA complex. The reliability was assessed with a goodness of fit of the model to the experimental data measured as root-mean-square error. Figure 2D shows the expression profiles of genes whose fit was 20% better when modeled with the complex HrdB–RbpA.

When analyzing the function of this group of genes, we found that the enrichment within the group was found for the ‘chromosomal replication’ group (just one gene SCO2064 DNA polymerase III subunit alpha of total eight genes), ‘adaptation’ (just 4 genes of 43, including two cold-shock proteins SCO3731 and SCO4505), ‘energy metabolism’ (3.2× more abundant with 25 genes of 189 total, comprising complete subunits of NADH dehydrogenase), ‘nucleotide biosynthesis’ (3.2×, 4 of 30 total, probably not significant); the group ‘ribosome constituents’ (13.8×, 38 of 67 total, including majority of ribosomal proteins) seems to be most important.

#### Genes controlled by the complex HrdB–RbpA only

(Supplementary File 1, sheets ‘hrdB or complex controlled’, ‘func for hrdBrbpA only’)

Among the 1694 genes that could be modeled by HrdB or the complex HrdB–RbpA, 41 genes that could be modeled exclusively by the complex HrdB–RbpA were found. Their expression profiles are shown in Figure 2E. Gene class enrichment analysis did not show any specific group of genes that would be characteristic of this group.

#### Genes not controlled neither by HrdB nor by the complex HrdB–RbpA

(Supplementary File 1, sheets ‘not controlled constitutively’, ‘not controlled at all’)

A total of 121 genes were found to be out of the control of any of the models used (Figure 2F). Analysis of the ontologies showed that the only enriched functional group was the ‘secondary metabolism’ group. In comparison to all the genes, the within group enrichment was quite strong (7×, 30 genes of total 277); most of them were from the polyketide synthesis pathway.

### sRNAs, tRNA, rRNAs

The streptomycete genome contains 105 known sRNA genes (32,33). Here, we identified 75 of them as being under HrdB control. These included T-box leader scr2076 and three miscellaneous RNAs—srp RNA (signal recognition particle RNA), *ssrA* gene for tmRNA and *rnpB* (probable ribonuclease P RNA), and further 17 conserved sRNAs that were identified in *Streptomyces avermitilis* and *Streptomyces venezuelae*.

Besides the sRNA, we also found 62 tRNAs within the HrdB regulon. As their genome possesses 63 tRNAs (4), HrdB was shown here to regulate almost the entire repertoire of tRNAs in *Streptomyces*.

At last, we identified all six sets of ribosomal RNA gene clusters in the streptomycetes genome (4) as members of the HrdB regulon; all of them exhibited a significant peak in ChIP-seq data (fold enrichment > 2) upstream of the gene for 16S rRNA and a smaller, less significant peak (fold enrichment around 1.8) upstream of the gene for 23S rRNA. Due to our statistic criteria, the latter were excluded by us from the HrdB regulon (all these RNAs are listed in Supplementary File 2).

### Promoter region binding motif search

#### Analysis of −10, −35 regions

**Figure 4.**
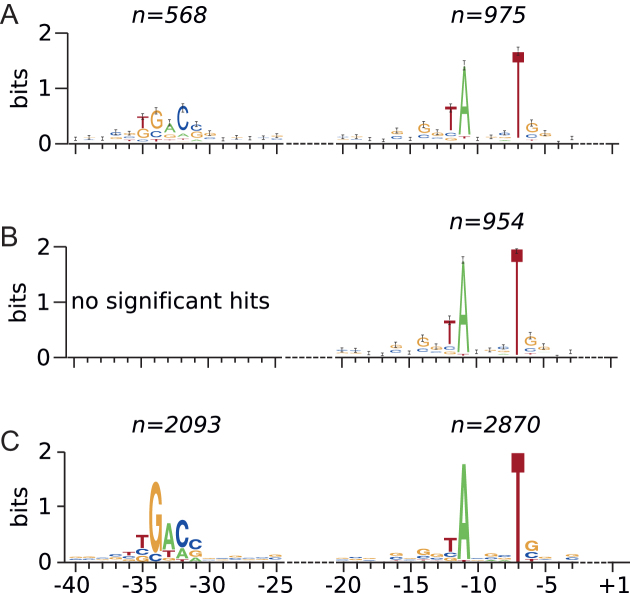
Comparison of found promoter binding motifs (**A** and **B**) with the published motif (**C**). N number of sites with *P*-value < 0.05. Horizontal axis represents consensual organisation of promoter region relative to TSS. The dashed line represents variable length region. (A) Motifs identified with method 1, the motif in −10 region (right) was identified at 93% sequences. (B) Motif for −10 region (right) obtained with method 2, the best motif for −35 region was present only in 40 sequences (4%) and we consider it as not significant and it is not shown. For −35 region (left) the motif was identified in 54% of sequences. (C) Image adopted from Jeong *et al*. The −10 motif is reported in 80% and the −35 motif in 59% of sequences. The motifs were drawn with Weblogo version 3.6.0.

For the genes with identified TSS we analyzed the −10 and −35 regions relative to TSS and compared the identified HrdB-binding motifs with general promoters found by an extensive transcriptional analysis (29). Figure 4 shows a comparison of the motifs found by method 1 and 2 (see ‘Materials and Methods’ section) with the motif published by Jeong *et al*. It is apparent that the motif found in the −10 region corresponds to that found by Jeong *et al*. very well, while the −35 region was less pronounced and was found significant only when method 1 was used. In order to find occurrence of the −10 motif among the genes not regulated by HrdB, we took the TSS dataset, filtered it for ‘Primary’ TSS and divided it into two parts, first that contained TSS that matched our Chip-seq identified sites and second that contained all TSS not included in the first dataset. We considered the second dataset NOT to be regulated by HrdB. For each TSS we extracted region from −20 to 0 upstream of each TSS. Then we used Fimo to scan extracted sequences (sites) for −10 motif occurrences. Number of sites with *P*-value < 0.05 were for HrdB-dependent genes 965 out of 1048 (92.08%), for those independent of HrdB were 1402 out of 1723 (81.37%), suggesting, in agreement with the findings of Jeong *et al*., that the −10 motif is quite general, not entirely specific for HrdB.

#### Analysis of −14, −13 regions

Our data suggest that the −35 element is lowly conserved across the HrdB regulon. This corresponds with the published data of genome-wide mapping of transcriptional start sites in another GC-rich *Actinomycete, M. tuberculosis*. It was shown that the majority of *M. tuberculosis* promoters lack the −35 elements (34). This observation was recently confirmed by *in vitro* and *in vivo* approaches where the impact of RbpA for transcription initiation at promoters lacking the −35 region was proved. The importance of guanosines at the position −13 and −14 was shown in *M. tuberculosis* as well (30). Supporting this fact, the crystallographic studies of mycobacterial transcription initiation complex revealed that RNAP is bound to −10 element by a sigma factor and this interaction is improved by RbpA binding to RNAP holoenzyme and to the upstream of −10 region, especially to the positions −13 and −14 (35). For these reasons, we performed a statistical analysis of guanosines in the mentioned positions (−14 and −13) of HrdB-dependent promoters to find out if they can play a similar role in *Streptomyces*.

Therefore, for the sites with the motif identified by the method 2 (Figure 4B) we examined positions −14 and −13 for the occurrence of G mono- and di-nucleotides. The region was divided into three groups according to occurrence of GG in the region - GG dinucleotide, only one G and no G (see Table 2). Table 2 also shows the occurrence of T at position −12 which was shown to be potentially significant. In order to enumerate the significance of occurrence of GG/G in the −14, −13 positions, we also calculated the frequency of GG dinucleotide occurrence in the whole genome and calculated the expected number of sites containing GG by chance in 954 sites (the same number as the number of analyzed sites). When the frequency of occurrence of GG in whole genome is 0.114 the proportion of expected sites in the 954 sites containing GG by chance is 109.The number of sites found in our data was about twice as high (see Table 2). Fischer test for occurrence of the GG motif in comparison with the HrdB independent TSS was significant (*P* = 0.00092). We can thus conclude that the occurrence of GG in the −14 and −13 positions is non-random and could play a role in the affinity of HrdB-RNAP holoenzyme to the promoter sequence in *Streptomyces*.

**Table 2.** Occurrence of GG/G in the −14 and −13 positions.

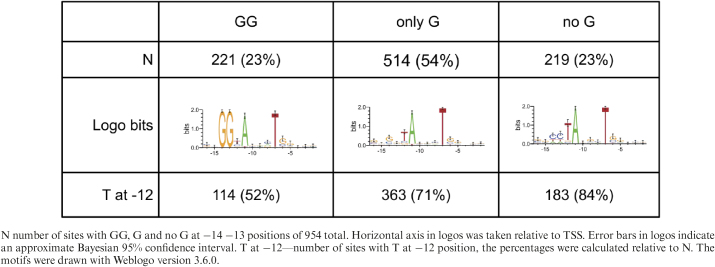

## DISCUSSION

In 1990 HrdB was shown to be essential for cell viability, as its deletion is lethal in *Streptomyces coelicolor* and it cannot be replaced by the other *hrd A, C* or *D* genes in its function (36). This finding was supported by the information that of the four *hrd* genes, only *hrdB* is present in all the investigated streptomycete species. Authors also suggest that HrdB could be a functional homolog of *E. coli*’s σ^70^. It was supposed that HrdB regulates the expression of housekeeping genes whose products are essential for the growth of streptomycetes.

However, due to the lethality of the cells after the deletion of *hrdB*, the native function of HrdB has not been investigated to date. A possible methodical solution to overcoming this difficulty is in tagging the protein of interest with a heterologous epitope. The epitope-tagging strategy eliminates the risk of cross-reactivity between the respective antibody and proteins with similar epitopes, which would be the case of various sigma factors in *Streptomyces*. Of the two existing approaches we used insertion of an epitope tag into the native chromosomal site of the protein-coding gene. Similar approach had been used before. For example, Petrone *et al*., (37) inserted FLAG3 tag to HilD protein to identify HilD regulon in *Salmonella enterica*. Similarly, Qian *et al*. (38) created a TAP-tagged GalR strain to identify GalR binding sites in *E. coli* and Stringer *et al*. (39) constructed a TAP-tagged AraC to map AraC binding sites in *E. coli*. For our purposes, we successfully adapted the method of PCR-targeting mutagenesis in *Streptomyces* (40) in order to introduce an epitope tag to the *hrdB* gene directly in the chromosome. Similarly, but independently, the method was presented by Kim *et al*. (41) who used tandem epitope tagging to insert a myc tag into the transcription factors ScbR and NdgR; this despite the fact that the longer repeated myc epitope tags have been shown to possibly affect the functionality of DNA-binding proteins (42,43). For this reason, we preferred the hemagglutinin (HA) tag. The HA tag, together with its specific and efficient commercially available antibody, exhibits valuable immunoprecipitation results while minimally affecting the target protein's function (43). Our created HA-HrdB strain was then subjected to ChIP-seq analysis, done in three repeats with a wild-type as a control. For this we used the same cultivation conditions as described in Nieselt *et al*. (23) in order to be able to exploit their gene expression database for the kinetics analysis.

### Model

The fact that a sigma factor is able to bind a promoter region *in vivo* does not always mean that the gene expression is actually switched on. Thus the σ^70^ subunit of *E. coli* may induce transcriptional pausing at promoter motifs (44,45). Also, our binding analysis was performed at one specific point, other conditions, or a different developmental stage, may involve other transcriptional regulators that might influence the HrdB-promoter affinities. The search for HrdB-regulated genes was performed with the assumption that HrdB is the principal regulator of mRNA accumulation during transcription. We have also modeled the situation when the gene expression rate is independent of the amount of HrdB altogether. As discussed below, it has been observed that RbpA forms a complex with HrdB and RNA polymerase enhancing expression of the regulated gene (11–13). We have therefore extended the model to incorporate the complex of HrdB–RbpA as the regulator as well. All three models (i.e. independent constitutive rate of expression, HrdB-dependent, and RbpA–HrdB complex-dependent) were utilized to prove or disprove the ability of HrdB to regulate the genes to whose promoters it bound, as discovered by the ChIP-seq experiment. The modeling of the found protein-coding genes revealed that HrdB was not strictly required only for 322 genes, as their expression rate was found to be independent of HrdB expression changes. Additional 121 genes could not be modeled with any of the three models. Here they are considered as false positives. We proved that the remaining 1694 genes could be controlled by HrdB, not only by the ChIP-seq, but also from the point of view of the expression kinetics.

### RbpA

The importance of the RbpA-HrdB complex is the reason why, as mentioned above, we included RbpA in the model. Looking at the expression profiles of HrdB and RbpA (Figure 3), the influence of RbpA is pronounced mainly in the later phases of growth when the RbpA profile peaks while the profile of HrdB slowly decays. The profiles of genes that reach higher levels in the later phase are thus better modeled with the addition of RbpA to the model, enhancing goodness of the fit to the given mRNA profile. The inclusion of RbpA as an additional regulator acting in complex with HrdB increased the model fidelity for 322 genes of the 1694 found to be controlled by HrdB. If less stringent criterion is used, the number of genes giving better modeling results for the complex of HrdB–RbpA increased to 579 genes. We therefore consider the activity of RbpA for the expression of HrdB regulated genes as substantial; this corresponds well with the previously described significance of the regulator in *Streptomyces* (14–17).

### Promoters

Based on transcriptional analyses and S1 nuclease mapping, only 20% of streptomycete promoters have similar sequences to those recognized by *E. coli* σ^70^ (6). As its regulon had not been identified earlier, the promoter specificity of HrdB sigma factor remained unclear. Several HrdB-binding sites had been published before (5,6,36,46). Using mostly *in vitro* transcription or by S1-nuclease mapping, these analyses revealed following genes: *furA, sigBp2, tuf3, chiD, gltBp1, ftsZp2, dagAp4, chiA, chiC, chiB, chiF, rrnDp2* and *rrnAp1*. From these, five genes (*tuf3, gltBp1, ftsZp2, rrnDp2, rrnAp1*) belonged to our HrdB regulon as well. The other genes were not transcribed by HrdB *in vivo* or they were transcribed by HrdB in other growth conditions or growth phases. Our dataset of HrdB-binding regions enabled us to search for specific binding motifs within the promoter sequences. For this, we compared the data we obtained using ChIP-seq with the published analysis of TSS (29) and we determined an HrdB–dependent promoter consensus sequence. Our search suggested that the majority of promoters in the HrdB regulon either lack the −35 element, or it has low conservation. On the other hand, the −10 element is highly conserved having thymine at −7 and adenine at −11 positions which perfectly correlates with motifs found by Jeong *et al*. (Figure 4). The shown similarity of the −10 region motifs between the published general motif search and our HrdB-dependent motif analysis supports the conclusion that HrdB is the predominant sigma factor for the vegetative growth phase. The conservation of the −10 element was also observed in *Mycobacterium*, where the majority of mycobacterial promoters lack the −35 element (34).

The previous studies revealed the importance of guanines that are located at the −14 and −13 positions upstream of the mycobacterial −10 element (30). We therefore performed a search to identify guanines at the mentioned positions. We analyzed the −14 or −13 position for 954 promoter sequences and at least a single guanine was identified in 77% of cases and in 23% of cases guanine was found in both positions. That suggests that promoter design in *Streptomyces* is very similar to that of *Mycobacterium*. Interestingly, search for guanines revealed the possibly important thymine at the −12 position. Percentage conservation of thymine at this position was increased by a decreasing number of guanines upstream. However, this hypothesis remains unconfirmed and will be subject to further investigation.

### Functional characterization of the HrdB regulon

#### HrdB targets involved in the morphological development

Within the HrdB regulon we identified genes that are involved in regulations of morphological differentiation and perhaps surprisingly in antibiotic production as well. Among them were *bld* genes, like *bldD* (SCO1489), *bldN* (SCO3323), *bldC* (SCO4091, a small DNA-binding protein related to MerR family of transcriptional activators (47)), *bldH/adpA* (SCO2792), and *bldB* (SCO5723, a key morphogenetic regulator responsible for the aerial hyphae formation (48)).

The main role of BldD during the vegetative growth is to repress genes responsible for further morphological differentiation and antibiotic production (49). Thus BldD is one of those regulators that maintain the vegetative phase of growth. Interestingly, within the HrdB regulon there are several genes (like those encoding for sigma factors σBldN and σWhiG) which have been previously shown to be controlled also by BldD (50) indicating substitutability of these two regulators.

Furthermore, other *whi* or *wbl* genes were found to belong to the HrdB regulon, like *whiB* (SCO3034), *wblE* (SCO5240), *wblH* (SCO6715) or *wblA* (SCO3579). Important developmental regulators belonging to the HrdB regulon were RNase III and AdpA. AdpA is a key regulator of morphological differentiation in *Streptomyces*, which was originally discovered in *Streptomyces griseus* (51,52). Less is known about its role in *S. coelicolor*, where its mutation induces a bald phenotype and a reduction in actinorhodin production (53). Recently it was found that a *cis-*antisense transcript regulates the expression of AdpA (54). The regulatory mechanism probably relies on an RNA duplex formation which is recognized by RNase III endoribonuclease. Other examples of HrdB-controlled pleiotropic regulators are *dasR* (SCO5231, a transcriptional repressor of antibiotic biosynthesis, nitrate metabolism, sugar transporters, etc. (55,56)) and a putative methyltransferase SCO2525 with a morphogenetic role (57). Other HrdB-regulated morphogenetic genes are those responsible for the crosswall formation and hyphae branching during the vegetative phase of growth. Proteins encoded by these genes are responsible for vegetative cell division. One such example is FtsZ, required for septation (58,59). Other examples are FtsI gene (SCO2090), essential in the process of peptidoglycan synthesis), SCO2968 (similar to FtsX in *M.tuberculosis*), FtsE (SCO2969), and FtsH-like protein (SCO3404).

#### Sigma factors within the HrdB regulon

In *Streptomyces*, a high number of sigma factors temporarily regulates gene expression depending on environmental cues or developmental state (60). A set of various sigma factors, anti-sigma factors and their antagonists was revealed in our analysis to be controlled by HrdB. Besides the above mentioned WhiG sigma factor, which is required for sporulation (50), there were other sigma factors such as HrdD (SCO3202), SigE (SCO3356, SCO5147), SigQ (SCO4908), BldN (SCO3323), SigI (SCO3068), SigK (SCO0632, SCO1723), anti-sigma factor RsbA (SCO0599) and anti-anti-sigma factor RsbB (SCO0598) within the regulon.

HrdD is a non-essential sigma factor that together with HrdA, HrdB and HrdC is homologous to sigma 70 subunit of *E. coli* (61). HrdD recognizes *in vitro* promoters of the pathway specific regulators actII-orfIV and redD controlling synthesis of actinorhodin and undecylprodigiosin (62). However, disruption of HrdD does not exhibit any obvious phenotypic defect (36). HrdD is very closely related to HrdB in its amino acid sequence and promoter specificity (63). Sigma factors HrdB and HrdD have almost identical 2.4 and 4.2 regions, which are responsible for binding sequence recognition (36,64) indicating overlapping or identical promoter specificities (61). Noteworthily, the expression of HrdD was previously shown to be partially dependent on SigE and SigR sigma factors (63). These sigma factors, mostly belong to the extracytoplasmic function (ECF) subfamily. Of these, SigE is required for normal cell wall synthesis (63). The other HrdB-dependent ECF sigma factors, SigI, SigH and probably also SigQ, are members of the streptomycete osmotic sensory system (65). Our previously published data of gene expression during spore germination revealed that expression of the HrdD, SigE, SigQ and SigI sigma factors are highly stimulated soon after the re-activation of spore metabolism (66). Thus, these sigma factors represent vegetative phase-specific regulators whose expression is dependent on HrdB, confirming again the vegetative-phase role of this essential sigma factor.

#### HrdB targets involved in energy metabolism

Our data showed that HrdB regulates the expression of nearly 50% of all genes involved in the energy metabolism of *Streptomyces*. More specifically, the HrdB regulon comprised 56% of genes from glycolysis (including 6-phosphofructokinase, triosephosphate isomerase, phosphoglycerate kinase, glyceraldehyde-3-phosphate dehydrogenase, and pyruvate kinase) and 65% of genes from the TCA cycle (including the key enzymes such as citrate synthase, succinyl-CoA synthetase, succinate dehydrogenases and others). No specific promoter sequence other than the conserved −10 sequence was found for the genes of glycolysis and TCA cycle, irrespective of the dependency of HrdB. Among others, citrate synthase CitA (SCO2736) has been previously shown to be crucial for maintaining the physiological balance of the cells. Its proper function is linked with cellular differentiation as its mutant loses abilities to form aerial mycelium and to produce antibiotics when grown on glucose (67). HrdB also participated in the expression of 46,5% genes from the electron transport system (ferredoxin-NADP-reductase, several types of cytochromes including cytochrome P450, cytochrome oxidase, oxidoreductases, NADH dehydrogenases, and electron transport proteins). Subsequently, the proton motive force is converted to adenosine triphosphate (ATP). The key enzyme, F0F1 ATPase, consists of a hydrophobic F0 component (formed from integral membrane proteins A, B and C) and a hydrophilic F1 component (formed from five subunits α, β, λ, δ and ϵ) (68). According to our results, the expression of the whole F0 component and at least two subunits (δ (SCO5370) and ϵ(SCO5374)) of the F1 component were also found to be controlled by HrdB.

Energy metabolism in *Streptomyces* is tightly regulated by a redox-sensing transcriptional repressor Rex (SCO3320). Rex protein responds to the cellular NADH/NAD+ levels and represses transcription of target operons *cydABCD* and *rex-hemACD*. Hence, Rex protein is considered as an essential regulator of aerobic metabolism. Moreover, the regulator also governs morphological differentiation by repressing *wblE* gene and affects avermectin production in *S. avermitilis* (69,70). Our Chip-seq experiments revealed that not only *rex* gene (SCO3320) (69) but also all the mentioned Rex-regulated genes are present in the HrdB regulon. Although *S. coelicolor* is usually classified as an obligate aerobe, it can use anaerobic respiration to generate energy and grow. Under oxygen limiting conditions, nitrate is utilized as an electron acceptor. This process is ensured by *nar* genes (71). Our ChIP-seq experiments revealed *nar2* operon, consisting of *narG2, narH2, narI2* and *narJ2* genes, as the members of the HrdB regulon. The findings mentioned above combined together demonstrate a general control of energy metabolism provided by HrdB as a response to oxygen quantity in the environment.

## CONCLUSION

The data and their consequent computational analysis presented here are the first attempt of its kind in the effort to discover the reasons behind HrdB’s function as the principal and indispensable sigma factor in *Streptomyces*. Other than the biological interpretation of the function of HrdB, the computational modeling, allowed us to associate the binding data with the actual expression of the incident genes and their regulator HrdB. The model and the computed parameters also allow for modeling changes in the expression of the target genes after artificial perturbations, performed by a computer, simulating a real experiment. This approach is a prerequisite for the construction of a general computational model for the control network of a studied organism.

Although the method was able to identify kinetically plausible interactions between a sigma factor and its target gene, it has some limitations. The model, in principle, can consider competition among sigma factors (or other factors), but they must be known. Without such independent knowledge, we are not able to add additional terms to the equations of the model. The model, therefore, is able to discover genes directly controlled by the sigma factor (here HrdB) and identify those, for which the regulation is more complex. The ChIP-seq data showed massive binding of HrdB–RNAP holoenzyme to HrdB-dependent promoters. These data suggested very strong affinity of HrdB to RNAP core. However, in *Streptomyces*, HrdB competes with other 65 sigma factors and the affinity of HrdB to the individual promoters plays a crucial role in its ability to control transcription of the particular gene. The competition of HrdB with other sigma factors for the binding site on RNAP core and the HrdB–RNAP holoenzyme binding affinity to HrdB-dependent promoters should be considered for more accurate analysis in future.

We show here that HrdB is indispensable because it delineates the key processes in the cells via expression control of genes responsible for central intermediary metabolism (gluconeogenesis, sulphur metabolism, fatty acid biosynthesis, pentose phosphate pathway and energy metabolism, amino acid metabolism, nucleotide biosynthesis and DNA replication), signal sensing and transduction (like PhoP-PhoR two component system or signal recognition particle complex), genes coding for other regulatory proteins and small RNAs (including tmRNA and RNase P RNA), pleiotropic developmental regulators and structural proteins involved in the morphogenesis, including cytoskeletal proteins and proteins involved in cell division, among other processes.

The number of identified essential genes found in its regulon prove the crucial role of HrdB in the life cycle of *Streptomyces*.

## DATA AVAILABILITY

Gene expression time series published by Nieselt (23): GEO accession number GSE18489. ChIP-seq data at ArrayExpress accession E-MTAB-6926

Genome sequence annotation file (.gtf) and the sequence file (fasta): Ensembl database ftp://ftp.ensemblgenomes.org/pub/bacteria/release-37/fasta/bacteria_0_collection/streptomyces_coelicolor_a3_2_/dna/.

Binding motifs: (29) in their supplement.

Source code for fitting the models: https://github.com/cas-bioinf/genexpi

## Supplementary Material

Supplementary Data
